# The rapid developments of membrane protein structure biology over the last two decades

**DOI:** 10.1186/s12915-023-01795-9

**Published:** 2023-12-29

**Authors:** Lan Guan

**Affiliations:** Department of Cell Physiology and Molecular Biophysics, Center for Membrane Protein Research, School of Medicine, Texas Tech University Health Sciences Center, Lubbock, TX 79424 USA

## Abstract

**Membrane protein research has flourished in the past 20 years. Exciting technological innovations in structural** **biology, including cryoEM single-particle analysis and AI-based protein structure prediction, such as AlphaFold 2, have largely revolutionized the field. The next decade promises great progress in understanding the critical roles of membrane transporters in health and disease and in the applications of novel drug design and development.**

## From biochemistry to molecular biology

Membrane transport proteins or transporters are a large group of integral proteins with multiple alpha-helices spanning cell membranes several times and folding in varied ways. Transporters play fundamental roles in physiological functions (such as nutrient absorption, signal transduction, and removal of toxic agents), and pathological processes (such as cancer development or pathogen invasion), by translocating specific substrates between the cell and its surroundings to maintain critical concentrations. Their cargo substrates include ions, small molecules (sugars, amino acids, or lipids), and macromolecules (proteins). Their specific location and functions in cell surfaces make them ideal drug targets to modify their functions for treatment or to hijack them for drug delivery. Although their hydrophobic surface and structural flexibility, which are the essential attributes for their biological functions, render them challenging to study, the field of membrane protein biology has excitingly progressed enormously in the past two decades. This comment article briefly summarizes the main technical developments that have accelerated this progress.

During the late 20th and early twenty-first centuries, advances in molecular biology promoted membrane transport research from biochemical analyses to the molecular level, to understand how transporters perform their functions. Most studies integrated a genetic approach with varied functional analysis, such as site-directed mutagenesis, Cys-scanning mutagenesis, and chemical modifications, e.g., to probe functionally critical residues, map the transmembrane topology, or explore the substrate-binding site [1]. The “membrane transport” community made much meaningful data available, however, data interpretations were somehow limited due to the fact that most transport proteins lacked high-resolution three-dimensional (3-D) structure information.

## Structural biology advanced the research of membrane transporters

In the past 20 years, and especially during the last 10 years, the field of membrane transport research has flourished and the high-resolution structures of many transporters have been experimentally determined. The number of solved structures of membrane proteins rapidly increased from zero or a few in several years to greater than 50 new unique structures yearly. These high-resolution 3-D structural breakthroughs largely advanced our understanding of how membrane proteins perform their specific work and the differences between diverse types of transporters. As a result, more new drugs targeting membrane proteins have been designed and synthesized.

Technological innovations in sample preparations and structure determination methods including computational methods and technology contributed mostly to this remarkable progress. X-ray crystallography has been the major tool for membrane protein structure determination until cryogenic electron microscopy (cryo-EM) single-particle analysis caught up in 2019 [2]. The resolution revolution of the cryo-EM technique significantly advanced the structure determination of membrane proteins to near-atomic resolutions. Moreover, growing well-diffracting crystals has been the major bottleneck for most membrane-protein structure determination by X-ray crystallography, and the cryo-EM technique skipped this time-consuming and tedious step. In addition, membrane protein production is often of low-yield, and the cryo-EM method does not require large amounts of protein samples. Furthermore, membrane transporters often populate multiple conformational states, and the cryo-EM image data processing can isolate more than one conformation. All these advances accelerated the membrane protein structure determination by cryo-EM [3], which enabled critical structural information to be available for biological research and drug development.

## Detergents/lipid nanodiscs

Cell membranes consist of a lipid bilayer with embedded membrane proteins. To study the membrane proteins, most techniques require the protein samples in solutions, not within the supporting membranes. The extraction of membrane proteins using amphiphilic molecules, such as detergents, from the hydrophobic membrane environments to generate membrane-mimic forms in solutions, is often an essential step before purification [4]. However, it is this essential step that often creates many problems. The extracted proteins are required to preserve their native fold and function in the absence of the native membrane environments. The stability of the detergent-solubilized protein samples is crucial for structure studies, especially for cryoEM single-particle reconstruction. The earlier developed nonanoic sugar-based detergents, such as undecylmaltoside (UDM), dodecylmaltoside (DDM), and octylglucoside (OG), are non-denaturing, which is critical to study membrane folding. Those membrane mimics have made significant contributions to the membrane protein structural and functional analyses. However, there is no magic detergent that can extract and maintain all membrane proteins due to the fact that membrane proteins are built in varied folds and architectures, and they interact with the heterogenic membrane lipids differently and dynamically. Most membrane proteins extracted using these earlier generations of detergents may not always be stable enough for structural determination, and some proteins are even not suitable for those detergents because extracted proteins are aggregated and functionally inactivated [4, 5].

Over the past two decades, a new generation of detergents has been invented, including the lauryl maltose neopentyl glycol (MNG) and the rigid hydrophobic-bearing detergents (GDN) [4]. Compared with DDM, those novel amphiphiles exhibit superior capability in preserving the stability of the extracted membrane proteins, hence they have greatly contributed to increasing the number of solved structures of membrane proteins, especially by cryo-EM single-particle analysis [4].

The invention of membrane scaffold protein-based lipid nanodiscs [6], or amphiphilic polymer-lipid particles (SMALP) provide excellent alternatives, as these generate nano-assemblies that contain the membrane proteins surrounded by a patch of native lipids, hence better preserving their native conformations and functions. Those nanodisc-like samples have also successfully facilitated cryo-EM single-particle reconstructive and membrane biophysical analyses.

## Nanobodies

The invention of varied tools to assist in membrane protein structure determination was another focus in the field and has also made tremendous progress in the past two decades. Nanobodies, small recombinant binding proteins derived from camelid single-chain antibodies, have been proven to be an ideal tool as crystallization chaperons [7]. Nanobodies can facilitate the crystallization of membrane proteins by decreasing protein dynamics or increasing surface mass for the crystal lattice contacts. Membrane proteins have often less-populated function-important states, which are challenging to study structurally and functionally. Conformation-specific nanobodies are very valuable tools for isolating specific conformations, enabling structural and functional analyses [8].

Various tools have also been created to increase the protein mass, as many transporters are too small for cryo-EM analysis. Most of these tools are based on fragment antigen-binding (Fab) fragments, such as the anti-nanobody Fab (NabFab) or fusions with a specific epitope for Fab recognition. Overall, those novel tools have enabled cryo-EM single-particle analysis of several groups of small transporters, often including drug targets, and have provided a wealth of critical structural information about transport mechanisms.

## Protein structure predictions with AlphaFold 2

In 2021, a remarkable but unexpected groundbreaking advance in structure biology emerged: the possibility of 3-D protein structure predictions directly from amino acid sequences, with the help of deep-learning-based AlphaFold 2 or RoseTTA programs [9]. The ability to predict 3-D protein structures, bypassing the costly and laborious experimental structure determination process has been a long-term “dream” for the computational biology community. All of a sudden, we can now just download predicted high-quality 3D structures of proteins of interest from the internet. This revolutionary achievement has far-reaching and lasting impacts on structural biology and biology in general. The ease of access and free availability of all AlphaFold 2 models will encourage non-structural biologists, such as cell biologists, geneticists, physiologists, and medicinal chemists to take advantage of rich structural information to accelerate life science research and drug development. The impact of prediction models on structure determination is multifaceted. The predicted models can be used for molecular replacement to bypass the bottleneck experimental “phasing problem” in macromolecular crystallography, and they can also be directly used for model building, especially for large complexes or assemblies.

It is worth stressing that this incredible achievement in protein prediction at high accuracies is the culmination of several decades-long efforts in the biology field. The free access to the massive numbers of depositions of experimentally determined protein structures in Protein Data Bank and protein sequence data of model organism proteomes in the Universal Protein Knowledgebase has been instrumental in this regard. That said, while protein structure prediction has a remarkably high-level accuracy in most cases, the predicted models are valuable hypotheses [10], and predictions on the details of ligand binding, protein-lipid interactions, protein oligemic states or complexes, and multiple conformational, including transient intermediates states, are still challenging. The current lack of related experimental data is one of the main reasons, highlighting the future directions of experimental structural biology.

## Summary

The remarkable progress in membrane protein structure biology, both experimentally and computationally over the past two decades, has generated enormously valuable structural knowledge, essential to our understanding of molecular mechanisms of solute transport and to advancements in drug development.

Structural models can enrich our knowledge of the mechanisms of protein actions but cannot predict protein function by themselves. Further research may emphasize the functional characterizations of membrane proteins whose role is still unknown since a battle of techniques and methods has been established for membrane protein studies. Experimental structural approaches will focus on more challenging areas, such as the structure determination of native cell membranes, protein complexes, and protein-lipid interactions, as well as elucidating transport regulatory mechanisms. The cutting-edge method of cryoelectron tomography (CryoET) enables visualization of protein structures in a native membrane environment instead of isolated membrane mimetic forms, which may not fully recapitulate in situ membrane biology. This innovative method is currently limited to large macromolecular complexes or assemblies but holds excellent potential for disclosing crucial structural information on membrane proteins, membrane protein complexes, and functional regulations in physiological environments. Future experimental studies will provide valuable datasets for training machine-learning algorithms to improve structure predictions and molecular dynamics calculations, eventually reducing the need for experimental approaches to confirm protein structure/function relationships, which is crucial for drug development.

## Data Availability

Not applicable.
